# Estimation of HIV incidence from analysis of HIV prevalence patterns by age and years since starting sex work among female sex workers in Zimbabwe

**DOI:** 10.1097/QAD.0000000000003198

**Published:** 2022-02-16

**Authors:** M Sanni Ali, Mariken De Wit, Sungai T Chabata, Sitholubuhle Magutshwa, Sithembile Musemburi, Jeffrey Dirawo, Brian Rice, Lucy Platt, Loveleen Bansi-Matharu, Harriet Jones, Tendayi Mharadze, Tarisai Chiyaka, Phillis Mushati, Owen Mugurungi, Raymond Yekeye, Amon Mpofu, Andrew N Phillips, Frances M Cowan, James R Hargreaves

**Affiliations:** 1 London School of Hygiene and Tropical Medicine, London, United Kingdom; 2 CeSHHAR, Harare, Zimbabwe; 3 University College London, London, United Kingdom; 4 Ministry of Health and Child Care, Harare, Zimbabwe; 5 National AIDS Council of Zimbabwe, Harare, Zimbabwe; 6 Liverpool School of Tropical Medicine, Liverpool, United Kingdom

**Keywords:** HIV, Female sex workers, Incidence, Prevalence, Respondent driven sampling, Zimbabwe

## Abstract

**Objectives:**

To estimate HIV incidence among female sex workers (FSW) in Zimbabwe: using HIV prevalence by age and number of years since started selling sex (YSSS).

**Design:**

We pooled data from FSW aged 18-39 participating in respondent-driven sampling surveys conducted in Zimbabwe between 2011-2017.

**Methods:**

For each year of age, we estimated: HIV prevalence (P_t_) and the change in HIV prevalence from the previous age (P_t_-P_t-1_). We then estimated the rate of new HIV infections during that year of age: I_t_=P_t_-P_t-1_/(1-P_t-1_), and calculated HIV incidence for 18-24 and 25-39 year-olds separately as the weighted average of I_t_. We estimated HIV incidence for FSW 1-5 years and 6-15 years since first selling sex using the same approach, and compared HIV prevalence among FSW first selling sex at their current age with the general population.

**Results:**

Among 9,906 women, 50.2% were HIV positive. Based on HIV prevalence increases by age, we estimated an HIV incidence of 6.3/100 person-years at risk (pyar) (95%CI 5.3,7.6) among 18-24 year-olds, and 3.3/100 pyar (95% CI 1.3,4.2) among 25-39 year-olds. Based on prevalence increases by YSSS, HIV incidence was 5.3/100 pyar (95% CI 4.3,8.5) between 1-5 years since first selling sex, and 2.1/100 pyar (95% CI -1.3, 7.2) between 6-15 years.

**Conclusions:**

Our analysis is consistent with very high HIV incidence among FSW in Zimbabwe, especially among those who are young and recently started selling sex. There is a critical need to engage young entrants into sex work in interventions that reduce their HIV risk.

## Introduction

Accelerating HIV incidence reduction is the most pressing concern for the global HIV community[1], especially in sub-Saharan Africa. However, across the region, a systematic approach to tracking epidemic trends among key populations, including female sex workers, is absent. Understanding of the rate of new infections among these populations is poor, undermining programmes ability to deploy data-informed implementation to reduce HIV incidence.

Female sex workers (FSW) in Zimbabwe have higher prevalence of HIV than women in the general population[2-4]. Zimbabwe’s National AIDS Strategic Plan identifies FSW and their clients as populations at increased risk of HIV[5-7]. Zimbabwe, like most countries in sub-Saharan Africa, relies on mathematical modelling to ascertain estimates of HIV incidence. One such model estimates that of new HIV infections in Zimbabwe since 2010, 70% (range 32% to 93%) were directly or indirectly attributable to transmission through sex work[8].

A range of survey methods, including respondent-driven sampling (RDS)[9] have been developed that can be used to estimate HIV prevalence in hidden populations (under several assumptions). HIV incidence can be estimated from such surveys using data from a recent infection testing algorithm[10]. In addition, demographic methods have been developed to estimate HIV incidence using cross-sectional data on HIV prevalence[11-15]. However, these methods have previously been applied to general population samples rather than FSW.

In this study, we pooled data from multiple surveys of FSW in Zimbabwe conducted over seven years. We explored HIV prevalence by age, and by years since started selling sex(YSSS). By adapting existing demographic methods, we estimated HIV incidence by age and by YSSS, and consider the potential biases, strengths and weaknesses of our approach in comparison with other approaches.

## Methods

### Setting

The Sisters with a Voice (“Sisters”) programme was established in Zimbabwe in 2009 on behalf of the Ministry of Health and Child Care(MoHCC) and the National AIDS Council[1]. By 2020, it operated nationally within 57 primary care clinics and provides comprehensive services in line with WHO guidelines[2]. In 10 of these clinics, generally found in larger towns, services are delivered in a fixed site, while in 47 of these clinics, services are delivered by a mobile team. Sisters provides free condoms and contraception, provider-initiated HIV testing and counselling, HIV self-testing and counselling (and secondary distribution of self-test kits for partners), syndromic management of sexually transmitted infections (STIs), health education and legal advice supported by a network of peer educators. Additionally, clinics provide long-acting reversible contraception (implants), referral for cervical cancer screening and on-site access to PrEP and, since 2020, anti-retroviral therapy and viral load testing.

Between 2011 and 2017, a “Sisters” affiliated research team conducted multiple RDS surveys in locations identified as sex work hot-spots across Zimbabwe [4,16-20]. Data were collected in RDS surveys of FSW in five separate research studies covering 21 sites in: 2011(3 sites), 2013 (14 sites), 2015(3 sites), 2016(14 sites) and 2017 three studies(4 sites+6 sites+2 sites) (Appendix Table 1A). The surveys in 2011 and 2015 were part of one study and were conducted in the same sites[16]. Similarly, the surveys in 2013 and 2016 were part of the same programmatic impact evaluation study and conducted in the same sites[17,18]. In all these surveys, FSW were eligible to participate if they had exchanged sex for money in the past 30 days, were aged ≥18 years, and had been living or working in the survey site for at least 6 months[16-18]. In addition, in 2017, an RDS survey was conducted among young women selling sex aged 18-24 years in 6 sites as part of an impact evaluation, and we included data in this analysis from young women recruited to this survey who self-identified as sex workers [19,20]. Also in 2017, RDS surveys were conducted in four sites including Zimbabwe’s two main cities as part of a National population size estimate[21]. Finally, in 2017, additional data were collected using RDS in 2 sites from young women selling sex aged 16-19 years[22]. In this study, young women were eligible if they had been working in the site for 30 days or more.

### Respondent driven sampling survey design

In each site, we conducted mapping followed by purposive selection of “seeds” representing a mix of ages, sex work types, and geographic locations[20]. We interviewed seeds, collected a blood sample for HIV testing and gave them two coupons to distribute to peers. Women receiving a coupon could attend an interview and were subsequently given two coupons for their peers. Five to seven iterations of this process (“waves”) were performed excluding the seeds. Participants were given US$5 compensation for their time, and US$2 for each referral who was eligible and recruited. Checks were included to ensure coupons were genuine and to avoid repeat participation. Interviewer‐administered questionnaire data were collected on tablet computers and included demographics, sex work, sexual behaviour, HIV prevention and care uptake and on personal network size for RDS adjustment. In all RDS surveys, participating women were offered point of care rapid HIV testing with return of results and counselling.

### Key variables

We pooled the data on HIV prevalence, site type (fixed/mobile site), age, and YSSS from all women participating in these surveys. The number of years since started selling sex was calculated as the difference between current age and the self-reported response to the question “how old were you when you first exchanged sex for gifts or money, i.e., when you first started selling sex”. In addition, we report the number of clients and condom use where this was collected.

### Statistical analysis

Our approach to RDS analyses have been described in detail elsewhere[16-20]. When reporting the specific studies, we followed the STROBE-RDS guidelines[23], first describing the population samples recruited at each site and assessing the evidence of bias in our operationalization of RDS[18,19]. We used the RDS‐II estimator[24] for analysis: dropping seed responses and weighting each woman in each site by the inverse of her network size, i.e., the number of other women she reports that she could have recruited. For this analysis, data were pooled across surveys and weighted using site‐normalized inverse degree weights.

Across the pooled data, we limited the analysis to FSW aged 18-39 years and those who had been selling sex for up to 15 years. Exclusion of older FSW and those who has been selling sex longer than 15 years was based on small sample sizes in these groups.

First, we described the socio-demographic and behavioural characteristics of the women in each of the surveys, and explored the relationship between age and YSSS. Second, we graphed and tabulated HIV prevalence by single years of age, for each study separately and in a pooled analysis. Age-specific HIV prevalence calculations were RDS-II weighted and confidence intervals were calculated.

Third, we estimated HIV incidence within two age groups. For each year of age, we calculated the prevalence increase since the previous year of age, and then divided this by (1– prevalence at the previous age): I_t_ = P_t_ - P_t-1_ / (1 - P_t-1_). For 18 year-old FSWs, we used the estimate of HIV prevalence among the 17 year old FSWs included in the AGSS survey to reflect P_t-1_. We calculated a weighted average of the estimates of I_t_ within the 18-24 year-old age group as an estimate of incidence for that group. Weighting was by the proportion of the sample of FSW in each year of age compared to the total within the age band. This was repeated for the 25-39 year-old age group. We also used bootstrapping to construct 95% confidence intervals for our estimated HIV incidence. To do so, for each age group (n=3000 for 18-24 year-olds, and n=5000, for 25-39 year-olds) and YSSS group (n=5000 among FSWs who reported 0-5 YSSS, and n=3000 among FSW who reported 6-15YSSS), we sampled 10,000 times, estimated the incidence, and used the 250th value and the 9750th value of the ranked incidence estimates as boundaries of the 95% confidence interval. The choice of the sample sizes considered the size of the original sample to produce reliable estimates of 95% confidence interval.

Fourth, we repeated the previous analysis approach to estimating HIV incidence, replacing age with YSSS. HIV incidence was estimated for two groups (1-5 and 6-15 YSSS), as a weighted average of the year-specific I_t_ estimates, and approximate confidence intervals calculated. FSW who had <1 year since they reported first selling sex were excluded from this stage of the analysis as it was not possible to calculate P_t-1_ for this group from the data. However, we also calculated HIV prevalence, and the average age (23 years), among the group of FSW reporting <1 YSSS, and compared this with HIV prevalence among women aged 20-24 years extracted from a national population-based survey conducted in 2015/16[26].

Fifth, and finally, we used logistic regression, with a quasi-binomial distribution, accounting for the RDS design to examine the association between age, YSSS and HIV prevalence. Since HIV incidence estimates appeared to differ in these age bands, we modelled HIV prevalence by year of age within the two age bands (18-24 years and 25-39 years) separately. HIV prevalence by YSSS was also modelled in the two groups (1-5 years and 6-15 years since start of selling sex) separately. We included site type (fixed or mobile), YSSS and age in these models with age modelled as a continuous variable. All analyses were conducted in R (Version 4.0.3)[27] using the Survey package for RDS data[28].

## Results

In total, 12,885 women were recruited to the RDS studies from 21 sites. We excluded those: with missing information on HIV test result and network size (n=440); who did not identify themselves as FSWs (n=760); and who were aged ≥ 40 years and/or who started selling sex ≥15 years ago. This resulted in 9,906 women included in our analyses (201 of whom were 17 years old or younger). The mean age of women included in the analysis was 26.9 years (SD=6.2). Women reported a mean age at start of sex work of 21.9 years (SD=5.3). Among 18-24 year-old FSW, median YSSS was 2 years (IQR=1-4), while among 25-39 year old sex workers it was 5 years (IQR= 3-9).

Across surveys 60.3-86.1% of FSW reported using condoms “all of the time” with clients. Number of (paying and non-paying) partners last month showed large fluctuation over the course of a month across surveys, due to factors such as pay day which results in an increase in sex work, and for SAPPH-IRe baseline and endline surveys[18], this variable was truncated at the average number of paying and no-paying sex partners in a month average number of paying and non-paying partners reported by FSW(300). Across the pooled data, 47.7% of respondent records were HIV positive (4,724/9,906), with this figure being much lower 24.3%(523/2156) in the two studies reporting on younger women only. Characteristics of women by survey is summarized in Table 1 and by age groups in Table 2.

**Figure 1:**
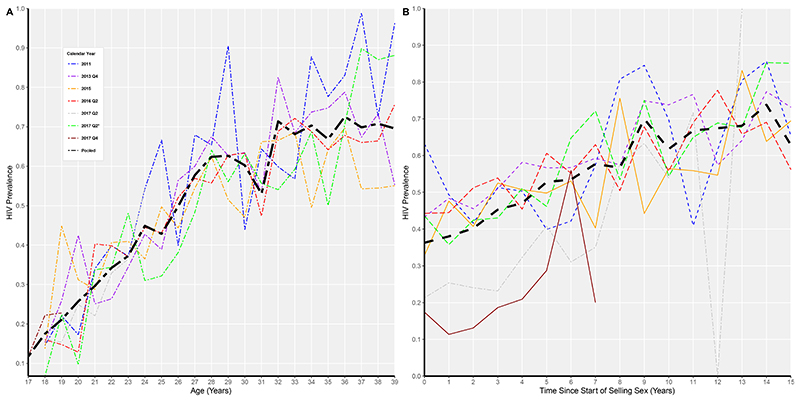
Prevalence by age and years since started selling sex in the different surveys (A and B) denoted by the calendar year of the survey. ^†^ The calendar years 2011 (GIZ 2011), 2013 Q4 (SAPHH-IRe baseline), 2015 (GIZ 2015), 2016 Q2 (SAPHH-IRe endline), "2017 Q2 (DREAMS), 2017 Q2* (Size Estimation), and 2017 Q4 (AGSS) refers to the surveys conducted in that specific year.

In each study, HIV prevalence increased sharply with age (Figure 1,Table 3), rising from 30.8% among 18-24 year-olds to 62.4% among 25-39 year-olds, and overall 50.2% in the pooled data (18-39 year olds). Data from the AGSS survey suggested an HIV prevalence of 11.7% among 17 year-olds, and thus we estimated 88.3% of FSW still to be at risk of acquiring HIV at age 18. We estimated annual HIV incidence to be 6.3 (95%CI 5.3, 7.6) per 100pyar among 18-24 year-olds, and 3.3 (95% CI 1.3, 4.2) per 100pyar among 25-39 year-olds(Table 3). In logistic regression, there was a strong association between HIV prevalence and age within each of the age bands: Odds Ratio (OR) for a single increased year of age 1.24 (1.17, 1.31) among 18-24 year-olds, and OR for a single year of age increase 1.08 (1.05, 1.10) among 25-39 year olds. After adjustment for YSSS, the association was similar, with adjusted odds ratios (aOR), 1.23 (1.17,1.31) and 1.07 (1.0,1.10), respectively(Table 3).

HIV prevalence also rose with YSSS, from 43.3% for women reporting 0-5 years since first selling sex to 61.4% among those reporting 6-15 years (Figure 1,Table 4). In the two studies recruiting only younger women, HIV prevalence was lower among those with fewer YSSS compared to the other studies(Figure 1). Among all FSW who reported starting selling sex at their current age (i.e. for whom YSSS<1) HIV prevalence was 36.3% (95% CI 28.1,44.4), and the mean age was 23 years. In the 2015 Zimbabwe national survey, HIV prevalence among 20-24 year olds was 8.2%[26].

We estimated annual HIV incidence as 5.3 (95% CI 4.3, 8.5) per 100pyar for women selling sex for 1-5 years, and 2.1(-1.3, 7.2) per 100pyar among those selling sex for 6-15 years. The difference in HIV prevalence between FSWs recruited to our RDS surveys who reported YSSS<1 and starting selling sex at their current age (mean=23) and women aged 20-24 years recruited to a national survey[26] and was 28.1%.

From the logistic regression model adjusting for site type, HIV prevalence was associated with each additional year since started selling sex, with OR 1.14(1.08,1.21) among those 1-5 YSSS, and 1.08(1.03, 1.14) for those 6-15 YSSS. However, after adjustment for age, the association disappeared, showing aOR 1.01(0.94,1.08) among FSW who have been selling sex for 1-5 years and aOR 1.01 (0.96,1.07) among those selling sex for 6-15 years (Table 4).

## Discussion

We pooled data from 9,906 respondents aged 16-39 participating in RDS surveys of female sex workers conducted in 21 locations in Zimbabwe between 2011 and 2017. We identified sharp increases in HIV prevalence with age. We estimated that these increases would be consistent with an underlying HIV incidence of 6.3 new infections per 100 pyar among 18-24 year-old FSW and 3.3 new infections per 100pyar among 25-39 year olds, under a range of assumptions discussed in more detail below. Using a more exploratory approach, we also saw rises in HIV prevalence over the number of years since women reported first selling sex. However, this was explained by the underlying association between age, YSSS and HIV prevalence. Women who reported starting selling sex at their current age had a much higher HIV prevalence than the estimated national prevalence of 8.1% among women 20-24 years old[26]. These data are consistent with a very high incidence of HIV infection among female sex workers in Zimbabwe, and with higher incidence among younger women and those in the early years of sex work.

Our study has several strengths. First, our analysis has a large sample size conducted from a hard-to-reach population in Zimbabwe, where data in this particular population are limited. The RDS surveys were distributed across the country so the findings reflect patterns among women selling sex in Zimbabwe. Indeed, the pattern of HIV prevalence by age and YSSS was strikingly similar across the different studies and over time. Second, all individual studies used robust and similar methods and their RDS diagnostics suggested minimal biases[18,19]. Third, our study used age-specific individual-level data on laboratory-based HIV test results and not self-reported HIV status (which is more prone to misclassification). The data were collected at a time of expanding access to antiretroviral therapy in Zimbabwe. A comprehensive government-led programme for ART supply has been available throughout the period of these RDS surveys, albeit under changing guidelines. The guidelines for ART were for people with CD4 less than <350 in 2011 but this changed in 2013 and ART was made available to FSW above this CD4 threshold from 2011. We have previously reported data on ART coverage from the same surveys used in this analysis. For example, in a recent paper we report that knowledge of HIV-positive status has increased from 48 to 78% between 2011 and 2016, and the prevalence of self-reported ART use among diagnosed women rose from 29% to 67% over this period (8).

Our approach to inferring HIV incidence from HIV prevalence by age and self-reported years since started selling sex among female sex workers participating in respondent driven sampling survey, is subject to a number of important limitations and should be interpreted carefully. We consider RDS surveys the most suitable approach to sampling given the nature of the FSW population, but we acknowledge that our samples are not truly random samples of the population. Over the years in relation to each of these surveys, we have done comprehensive diagnostic testing as recommended in the literature – and although it is not possible to prove representativeness, we have been not identified evidence of significant biases.

HIV prevalence increases by year of age can only be interpreted as reflective of HIV incidence if a number of assumptions hold: 1) age-specific HIV incidence is stable over time, 2) age-specific mortality rates are similar among HIV positive and HIV negative women, and 3) the rate at which women enter and leave sex work in each study location is similar and not affected by HIV status. Our analysis was not able to account for possible differential risk of entering or leaving sex work (i.e. the probability of entering or leaving sex work being dependent on HIV status), or risk of death between HIV positive and negative women. However, it is plausible that these effects may be modest in the context of universal ART access and relatively high levels of use, and particularly so among younger women. Inferring HIV incidence from patterns of HIV prevalence with age has been used before in general population samples where mortality and migration were the major threats to valid interpretation [13-15]. Nevertheless, we recognise that it is plausible women who are HIV positive may leave sex work at a higher rate that those who are HIV negative, particularly at older ages, the impact of which would be to bias our method toward an underestimation of HIV incidence. We were unable to identify data that would allow us to accurately correct our estimate for this possibility but would encourage future analyses to consider this is it proved feasible.

An additional feature of our study is that women enter and leave sex work over time. Previous work has suggested that the median duration of sex work is 6 years globally[29]. Reflecting our interest in the risk of HIV infection during periods of sex work, we implemented an additional exploratory approach to the analysis, bringing in data on self-reported number of years since women first sold sex. A limitation to these analyses is that our estimates will be biased if YSSS is estimated inaccurately, which is plausible given the sensitive nature of the data and, for some women, long recall periods. However, a small inaccuracy in estimations for this group is unlikely to have a large impact on our results. Additionally, if women cycle into and out of sex work this may limit the validity of the estimation. Interestingly in our analysis, after adjustment for age, the association between years since started selling sex and HIV prevalence was attenuated.

A further dimension of interest in the relation between HIV prevalence and YSSS was that HIV prevalence among women who reported that they had started selling sex for less than a year at their current age was 28.1% higher than among women of the same age group nationally. These women might be thought of as the source population from among which women enter sex work. This estimation may also be limited by misreport of YSSS. However, two other dynamics of interest are also compatible with this finding. First, women with prevalent HIV infection may be preferentially selected into sex work. Many studies show that the prevalence of widowing and divorce/separation is high among FSW in Africa[18], and that this is associated with elevated HIV prevalence. An alternative explanation is that women may experience a very high risk of new HIV infection during their first months of engagement in sex work. Indeed, if all of this difference in prevalence were explained by new infections in the first year of sex work, this would represent an incredibly high incidence rate of approximately 30 new infections per 100pyar. Similarly, in Tanzania gold mines, much higher rates of HIV infection were reported in the first year after the mine opening among women in the community [30]. Women in the RDS surveys we undertook needed to report having lived for at least 6 months in the site where they were recruited, except two sites (Harare and Bulawayo) where they reported to have lived at least 30 days, and engaged in sex work in the past 30 days. Client burden is high, and previous studies have suggested that young, new entrants to sex work may be particularly vulnerable and with little ability to negotiate safe sex with clients[31,32]. While relatively little data are available on the prevalence of unsuppressed HIV infection among the clients of sex workers, it is plausible that this may also be high. Rates of viral suppression in males is age dependent with very low rates among with younger men[26].

There are relatively few direct estimates of HIV incidence for FSW populations in Africa[33] Those that do exist are mostly for cohorts recruited from clinics, bars or sex work hot spots in East Africa which have reported incidence rates between 13.1/100pyar (95%CIs 11.02,15.64) in 1993-1997 in Mombasa, Kenya[34], 4.5/100pyar in Nairobi, Kenya in 2000-2002 [35], and 2.2/100pyar (1.60,3.1) in Nairobi, Kenya between 2008-2011[36]. Another study in Benin reported HIV incidence of 0.8/100 person-years among PREP participants[37]. Our findings are also slightly lower than another study in South Africa which recruited a cohort of 245 high risk women (the majority of whom self-identified as sex workers) between 2004 and 2005 and found an incidence rate of 7.2 (95% Cl: 4.5,9.8) per 100pyar[38].

In addition, HIV incidence has previously been estimated among FSW in Zimbabwe using a number of different approaches. A previous study by our group[4] measured the rate of seroconversion from clinic HIV test data among sex worker programme attendees in Zimbabwe, estimating incidence at 9.8 per 100pyar between 2009 and 2014. In the same programme platform in 2016 we found that 33/313 (10.5%) women who tested positive were classified as recently infected, with higher recent infection rates among younger women [39]. During an impact evaluation of DREAMS, baseline data from which were included in this analysis, a cohort of young women selling sex recruited by RDS were followed over approximately 24 months in 6 sites. HIV incidence varied from 2.7 to 7.1/100pyar, and was non-significantly lower in DREAMS towns than non-DREAMS towns after adjustment[40]. Other studies showed that recruitment into a cohort can ‘interfere with’ risk of HIV infection and may result in poorer measure of incidence in wider population [35,41].

## Conclusion

These data point to very high rates of new HIV infection among female sex workers, especially among those who are young and new entrants in to selling sex. There is a critical need to strengthen and sustain existing HIV prevention and treatment programmes for female sex workers, and to develop strategies that engage young women new to sex work to reduce their risk of acquiring HIV infection and ensuring that if infected they are rapidly identified and started on treatment. There is also an ongoing need to strengthen data collection and analysis to inform estimates of HIV incidence in this group.

## Supplementary Material

table Appendix Table 1a

## Figures and Tables

**Table 1:** Characteristics of women participating in the surveys by calendar year of survey (N=9906)

	Surveys including female sex workers aged 18-39					Surveys of younger women	
	GIZ 2011 (N=656)	SAPPH_Ire baseline 2013 (N=1872)	GIZ 2015 (N=710)	SAPPH_Ire endline 2016 (N=2271)	Size Estimation 2017 (N=2241)	AGSS (16-19 year olds) 2017 (N=495)	DREAMS (18-24 year olds) 2017 (N=1661)
**Age (Years)**							
Mean (SD)	27.7 (5.6)	28.4 (5.6)	29.1 (5.8)	29.4 (5.4)	28.6 (5.7)	17.7 (1.1)	21.1 (2)
**Age when first sold sex (Years)**							
Mean (SD)	22.2 (5.4)	23.1 (5.0)	22.9 (5.3)	23.4 (5.1)	23.3 (5.2)	15.7 (1.4)	18 (2.3)
**Years since started selling sex (Years)**							
Median [IQR]	4 [2-8]	4 [2-7]	4[2-8]	4[3-8]	4[2-7]	2 [1-3]	3 [1-4]
**Marital Status**							
Single/never married	195 (29.7%)	387 (20.7%)	194 (27.3%)	391 (17.2%)	511 (22.8%)	386 (78.0%)	865 (52.1%)
Married/living together	11 (1.7%)	11 (0.6%)	3 (0.4%)	35 (1.5%)	30 (1.3%)	3 (0.6%)	23 (1.4%)
Divorced/separated	364 (55.5%)	1234 (65.9%)	441 (62.1%)	1571(69.2%)	1507 (67.2%)	104 (21.0%)	751 (45.2%)
Widowed	86 (13.1%)	240 (12.8%)	68 (9.6%)	274 (12.1%)	193 (8.6%)	2 (0.4%)	22 (1.3%)
**Number of partners last month** ^††^							
Median [IQR]	10 [5-20]	64.5 [16-300]	60 [2-140]	20 [10-40]	30 [15-60]	15 [6-30]	7 [4-15]
**Number of clients last week**							
Median [IQR]	-	5 [3-10]	6 [3-15]	5 [3-10]	7 [4-15]	12 [5-26]	-
**Condom use last sex with partner** ^†^							
No	-	454 (43.1%)	157 (38.7%)	643 (47.5%)	605 (53.0%)	-	-
Yes	-	597 (56.8%)	249 (61.3%)	711 (52.5%)	536 (47.0%)	-	-
**Condom use last sex with client** ^†^							
No	35 (5.6%)	61 (3.9%)	29 (4.1%)	68 (3.0%)	89 (4.0%)	-	-
Yes	595 (94.4%)	1496 (96.1%)	676 (95.9%)	2196 (97.0%)	2150 (96.0%)	-	-
**Condom with client, last month**							
Always - 100% of time	-	1141 (66.5%)	490 (71.6%)	1272 (60.3%)	1930 (86.1%)	-	-
Mostly about 75%	-	198 (11.5%)	103 (15.1%)	212 (10.0%)	160 (7.1%)	-	-
Sometimes about 50%	-	108 (6.3%)	22 (3.2%)	128 (6.1%)	103 (4.6%)	-	-
Rarely 25% of the time	-	45 (2.6%)	18 (2.6%)	89 (4.2%)	23 (1.0%)	-	-
Never	-	223 (13.0%)	51 (7.5%)	409 (19.4%)	25 (1.1%)	-	-
**HIV Test result**							
Negative	282 (43.0%)	829 (44.3%)	337 (47.5%)	1027 (45.2%)	1074 (47.9%)	412 (83.2%)	1221 (73.5%)
Positive	374 (57.0%)	1043 (55.7%)	373 (52.5%)	1244 (54.8%)	1167 (52.1%)	83 (16.8%)	440 (26.5%)

† Condom use in the last month with sex partner and client are both calculated among those FSW who reported having sex partner and clients, respectively.

†† Number of partners last month, refers to the question “How many people have you had vaginal sex with in the last month (including paying and non-paying partners)”, showed large fluctuation over the course of a month due to factors such as pay day which results in an increase in sex work, and was truncated at the mean number of paying and no-paying sex partners in a month (300, i.e. 60 per week).

**Table 2:** Characteristics of women participating in the surveys by age group (N=9705, excluding 17 years or younger)

	Age Groups		
	18-24 Years (N=4024)	25-39 Years (N=5681)	Overall (N=9705)
**Age (years)**			
Mean (SD)	21.2 (2)	31.4 (4.2)	26.9 (6.2)
**Age when first sold sex (Years)**			
Mean (SD)	18.3 (2.3)	24.7 (5.0)	21.9 (5.3)
**Years since started selling sex (Years)**			
Median [IQR]	3 [2-6]	4 [2-7]	4 [2-7]
**Marital Status**			
Single/never married	1941 (48.2%)	810 (14.3%)	2929 (29.6%)
Married/living together	42 (1.0%)	72 (1.3%)	116 (1.2%)
Divorced/separated	1961 (48.7%)	3990 (70.2%)	5972 (60.3%)
Widowed	79 (2.0%)	806 (14.2%)	885 (8.9%)
**Number of partners last month**			
Median [IQR]	20 [7-50]	20 [10-60]	20 [8-60]
**Number of clients last week** ^†^			
Median [IQR]	7 [4-15]	6 [3-12]	6 [3-14]
**Condom use last sex with steady partner** ^†^			
No	532 (49.4%)	1327 (46.2%)	1859 (47.0%)
Yes	545 (50.6%)	1548 (53.8%)	2093 (53.0%)
**Condom use last sex with client**			
No	78 (4.0%)	204 (3.8%)	282 (3.8%)
Yes	1893 (96.0%)	5220 (96.2%)	7113 (96.2%)
**Condom with client, last month**			
Always - 100% of time	1239 (70.0%)	3594 (72.2%)	4833 (71.6%)
Mostly about 75%	187 (10.6%)	486 (9.8%)	673 (10.0%)
Sometimes about 50%	112 (6.3%)	249 (5.0%)	361 (5.3%)
Rarely 25% of the time	48 (2.7%)	127 (2.5%)	175 (2.6%)
Never	183 (10.3%)	525 (10.5%)	708 (10.5%)
**HIV Test result**			
Negative	2827 (70.3%)	2171 (38.2%)	5182 (52.3%)
Positive	1197 (29.7%)	3510 (61.8%)	4724 (47.7%)

† Condom use in the last month with sex partner and client are both calculated among those FSW who reported having a sex partner(s) and a client(s) respectively.

**Table 3:** Estimates of HIV prevalence and incidence by age (n= Number of HIV positive FSW and, N = total FSW in each age band).

Age, years	n	N	At risk fraction at start of period (%)	Prevalence % (95% CI)	Delta Prevalence (%)	Delta Prev/ At risk	Estimated annual rate of new infections in age bands (%)^†^	Site-type adjusted Odds Ratio for HIV Prevalence increase by single year of age (95 % CI) ^‡§^	Site-type and YSSS adjusted Odds Ratio for HIV Prevalence increase by single year of age (95% CI)^¶¥^
16 ^f^	1	81	100	0.9 (-0.9, 2.9)	1.0	0.99	-	-	-
17 ^f^	16	120	99.0	11.7 (5.3, 18.0)	10.8	10.7	-	-	-
18	89	502	88.3	17.4 (13.1, 21.8)	5.8	6.5	6.3 (5.3, 7.6)	1.24 (1.17, 1.31)	1.23 (1.17, 1.31)
19	111	582	82.5	21.1 (16.3, 25.8)	3.6	4.4			
20	119	427	78.9	25.7 (19.4, 31.9)	4.6	5.9			
21	142	531	74.3	29.6 (23.7, 35.5)	3.9	5.3			
22	212	641	70.3	34.2 (28.8, 39.5)	4.6	6.5			
23	297	810	65.8	37.2 (32.1, 42.4)	3.0	4.6			
24	227	531	62.7	44.8 (38.1, 51.6)	7.6	12.2			
25	175	383	55.1	42.8 (35.2, 50.4)	-2.0	-3.7	3.3 (1.3, 4.2)	1.08 (1.05, 1.10)	1.07 (1.0,1.10)
26	204	427	57.2	49.8 (42.5, 57.1)	7.0	12.3			
27	242	432	50.1	57.7 (50.0, 65.4)	7.9	15.7			
28	242	417	42.3	62.3 (55.6, 69.2)	4.7	11.0			
29	269	468	37.6	62.6 (55.3, 70.0)	0.3	0.7			
30	282	447	37.4	60.2 (52.6, 67.8)	-2.4	-6.5			
31	251	430	39.7	53.0 (45.5, 60.5)	-7.2	-18.2			
32	288	453	47.0	71.3 (65.3, 77.4)	18.4	39.1			
33	269	411	28.6	68.1 (61.5, 74.7)	-3.3	-11.4			
34	261	395	31.9	70.3 (63.4, 77.2)	2.2	6.9			
35	204	301	29.7	66.8 (58.8, 74.9)	-3.5	-11.7			
36	202	284	33.1	72.5 (64.3, 80.7)	5.7	17.2			
37	244	331	27.4	69.8 (61.7, 78.0)	-2.7	-9.9			
38	213	289	30.1	70.7 (62.3, 79.2)	0.9	3.1			
39	164	213	29.2	69.5 (57.7, 81.4)	-1.2	-4.1			

† For incidence calculation within age bands, weighted average of incidence was used, and the overall estimate of incidence across ages 18-39 was 4.6%.

‡ The model was fitted using data with the specified age bands separately (18-24 years and 25-39 years).

§ When the same model was fitted using the full data (ages 18-39 years) and adjusting for site type (mobile/fixed), OR and 95%CIs were 1.08 (1.05,1.10).

¶ When adjusted for YSSS, YSSS was used as a continuous variable in the model for both age strata.

¥ When the same model was fitted using the full data (age group 18-19, adjusting for site type and YSSS), OR and 95%CIs were 1.07 (1.05,1.10).

f the first two rows were only descriptive purpose and were not included in the calculation of incidence.

**Table 4:** Estimates of HIV prevalence and incidence by years since started selling sex (YSSS) (n= Number of HIV positive FSW and, N = total FSW in each YSSS band).

YSSS	n	N	At risk fraction at start of period %	Prevalence (95% CI), %	Delta Prevalence	Delta Prevalence / At risk %	Estimated annual rate of new infections in YSSS bands^†^	Site-type adjusted Odds Ratio for HIV Prevalence increase by single year increase in YSSS (95 % CI)^‡ §^	Site-type and age adjusted Odds Ratio for HIV Prevalence increase by single year single year increase in YSSS (95% CI)^¶¥^
N/A	147	1817	-	8.1^¢^	-	-	-	-	-
0^f^	81	279	91.8	36.3 (28.1, 44.4)	28.1	30.6	-	-	-
1^f^	457	1352	63.7	38.0 (33.9, 42.1)	1.7	2.7	5.3 (4.3, 8.5)	1.14 (1.08,1.21)	1.01 (0.94,1.08)
2	637	1719	62	40.3 (36.6, 43.7)	2.2	3.5			
3	636	1483	59.8	45.3 (41.3, 49.3)	5.1	8.5			
4	491	1119	54.7	47.1 (42.6, 51.5)	1.8	3.2			
5	404	778	53.0	52.8 (47.4, 58.1)	5.7	10.8			
6	314	602	47.2	53.5 (46.9, 60.1)	0.7	1.5	2.1 (-1.3, 7.2)	1.08(1.03,1.14)	1.01 (0.96,1.07)
7	267	488	46.5	57.7 (50.7, 64.7)	4.2	9.1			
8	221	355	42.3	56.7 (48.5, 64.8)	-1.0	-2.4			
9	205	293	43.3	69.9 (61.4, 78.4)	13.2	30.6			
10	192	299	30.1	61.8 (53.3, 70.3)	-8.2	-27.2			
11	143	210	38.2	66.8 (55.9, 77.7)	5.0	13.2			
12	121	174	33.2	67.4 (56.1, 78.8)	0.6	1.9			
13	92	137	32.5	68.1 (56.1, 80.1)	0.7	2.2			
14	93	126	31.9	73.8 (63.3, 84.3)	5.7	17.9			
15	86	119	26.2	62.9 (46.5, 79.1)	-11	-41.8			

† For incidence calculation within YSSS bands, weighted average of incidence was used, and the overall estimate of incidence across YSSS 1-15 years was 4.4%.

‡ The model was fitted using data with the specified YSSS bands separately (1-5 years and 6-15 years),

§ When the same model was fitted using the full data (YSSS 1-15 years) and adjusting for site type (mobile/fixed), OR and 95%CIs were 1.11 (1.08,1.14).

¶ Age was used as a continuous variable in all the models.

¥ When the same model was fitted using the full data (YSSS groups 1-15 years, adjusting for site type and age), OR and 95%CIs were 1.02 (0.99,1.04).

f The first row was only descriptive purpose and was not included in the calculation of incidence.

¢ This figure comes from ZIMPHIA 2015-2016 [24] and represents the HIV prevalence among women aged 20-24 years in Zimbabwe nationally in 2015/16.
