# Supplementary material for: Estimation of HIV incidence from analysis of HIV prevalence patterns by age and years since starting sex work among female sex workers in Zimbabwe
Source: AIDS. Author manuscript; Available in PMC 2025 Oct 9. (PMC7618220; doi:10.1097/QAD.0000000000003198)
Supplement: table Appendix Table 1a [file EMS208731-supplement-table_Appendix_Table_1a.docx]

**Supplementary Material**

**Table 1A: RDS Survey location and year of Survey**

| Sites | 2011 | 2013 Q4 | 2015 | 2016 Q2 | 2017 Q2 (DR) | 2017 Q2 (SE) | 2017 Q4 |
| --- | --- | --- | --- | --- | --- | --- | --- |
| 1 |  | Bindura |  | Bindura |  |  |  |
| 2 |  |  |  |  | Bulawayo | Bulawayo | Bulawayo |
| 3 |  | Chinhoyi |  | Chinhoyi | Chinhoyi |  |  |
| 4 |  | Chipinge |  | Chipinge |  |  |  |
| 5 |  | Chivhu |  | Chivhu |  |  |  |
| 6 |  | Gutu |  | Gutu |  |  |  |
| 7 |  | Gwanda |  | Gwanda |  |  |  |
| 8 |  |  |  |  |  | Harare | Harare |
| 9 | Hwange | Hwange | Hwange | Hwange |  |  |  |
| 10 |  | Juru |  | Juru |  |  |  |
| 11 |  | Kadoma |  | Kadoma |  |  |  |
| 12 |  | Kariba |  | Kariba |  |  |  |
| 13 |  |  |  |  | Karoi |  |  |
| 14 |  |  |  |  | Kwekwe |  |  |
| 15 |  | Magunje |  | Magunje |  |  |  |
| 16 |  | Marondera |  | Marondera |  |  |  |
| 17 | Mutare |  | Mutare |  | Mutare |  |  |
| 18 |  | Ngundu |  | Ngundu |  |  |  |
| 19 |  |  |  |  |  | Shamva |  |
| 20 | Vic Falls |  | Vic Falls |  |  |  |  |
| 21 |  | Zvishavane |  | Zvishavane | Zvishavane |  |  |
